# Biological small-calibre tissue engineered blood vessels developed by electrospinning and in-body tissue architecture

**DOI:** 10.1007/s10856-022-06689-w

**Published:** 2022-09-30

**Authors:** Zhixiang Su, Yuehao Xing, Fei Wang, Zeqin Xu, Yongquan Gu

**Affiliations:** 1 Vascular Surgery Department, Xuanwu Hospital, Capital Medical University, 100053 Beijing, China; 2 Department of Cardiovascular Surgery, Beijing Children’s Hospital, National Center for Children’s Health, Capital Medical University, 100045 Beijing, China

## Abstract

There are no suitable methods to develop the small-calibre tissue-engineered blood vessels (TEBVs) that can be widely used in the clinic. In this study, we developed a new method that combines electrospinning and in-body tissue architecture(iBTA) to develop small-calibre TEBVs. Electrospinning imparted mechanical properties to the TEBVs, and the iBTA imparted biological properties to the TEBVs. The hybrid fibres of PLCL (poly(L-lactic-co-ε-caprolactone) and PU (Polyurethane) were obtained by electrospinning, and the fibre scaffolds were then implanted subcutaneously in the abdominal area of the rabbit (as an in vivo bioreactor). The biotubes were harvested after four weeks. The mechanical properties of the biotubes were most similar to those of the native rabbit aorta. Biotubes and the PLCL/PU vascular scaffolds were implanted into the rabbit carotid artery. The biotube exhibited a better patency rate and certain remodelling ability in the rabbit model, which indicated the potential use of this hybridization method to develop small-calibre TEBVs.

**Figure:**
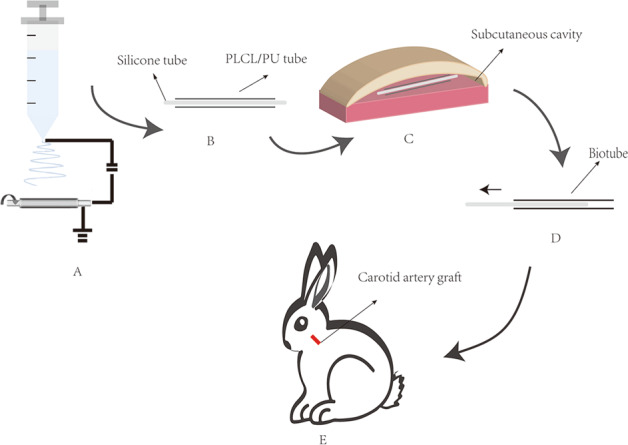
Sketch map of developing the biotube. The vascular scaffolds were prepared by electrospinning (A). Silicone tube was used as the core, and the vascular scaffold was used as the shell (B). The vascular scaffold and silicone tube were implanted subcutaneously in the abdominal area of the rabbit (C). The biotube was extruded from the silicone tube after 4 weeks ofembedding (D). The biotube was implanted for the rabbit carotid artery (E).

Sketch map of developing the biotube. The vascular scaffolds were prepared by electrospinning (A). Silicone tube was used as the core, and the vascular scaffold was used as the shell (B). The vascular scaffold and silicone tube were implanted subcutaneously in the abdominal area of the rabbit (C). The biotube was extruded from the silicone tube after 4 weeks ofembedding (D). The biotube was implanted for the rabbit carotid artery (E).

## Introduction

In cardiovascular diseases (CVDs), traumatic injury, and end-stage renal diseases (ESRDs), the need for vascular grafts and vascular access is significant and increasing, especially for small-calibre blood vessels (<6 mm). To date, autologous blood vessels are still the ideal material for small-calibre vascular grafts because of their superior patency [1]. However, autologous grafts still have limitations, such as poor quality (excessively thick or thin), harvesting invasiveness, and unavailability (due to systemic atherosclerosis or the vessels having already been harvested) [2]. Therefore, practical and alternative small-calibre tissue-engineered blood vessels (TEBVs) are in high demand.

Many approaches for developing tissue-engineered blood vessels have been described [3], such as decellularization, electrospinning, lyophilization, 3D bioprinting [4] and in-body tissue architecture (iBTA) [5]. Acute thrombosis, mechanical failure, intimal hyperplasia, infection and growth potential are the main challenges associated with these approaches [6]. To meet these challenges, we created a new method that combined electrospinning and iBTA to develop the small-calibre TEBVs in this study.

Electrospinning can fabricate fibrous scaffolds with fibre diameters ranging from nanometres to micrometres, which have physical properties close to those of the natural extracellular matrix (ECM). The electrospun fibrous scaffold has large surface areas, pore-interconnectivity and high porosity and provides suitable surface sites for the cells to adhere, proliferate, and grow [7]. The main limitation of electrospinning synthetic vasculature is the lack of biological properties, which can result in incomplete endothelization, stenosis, thrombosis, and limited function of the vascular graft [4]. Utilizing the iBTA can enhance the biological properties [5]. The method of using the host as an in vivo bioreactor is not new; for example, Sparks applied autologous tissue capsules as vascular grafts in the 1960s. These grafts were briefly used in clinical applications, but the primary problem related to them was the lack of sufficient mechanical properties [8]. Through tailoring the foreign body response (by modulating the biomaterial characteristics, implantation period, and host features) [5] and designing novel moulds [9], the in vivo TEBVs show excellent potential. The in vivo fibrous capsule is rich in collagen, (myo-) fibroblasts, and one or two layers of macrophages and multinucleated foreign body giant cells, which are components of the ECM and have outstanding biological properties [5]. Based on these previous studies, we first developed TEBVs by electrospinning to obtain the mechanical properties and then implanted them into rabbits subcutaneously for four weeks to obtain the biological properties. Here, we describe this hybridization method in the rabbit model and the situation of carotid artery allograft.

## Materials and methods

### Materials

Poly (L-lactic-co-ε-caprolactone) (PLCL, L-lactic acid/e-caprolactone 50:50, IV:3.2 dl/g), Jinan Daigang Biomaterial Co., Ltd. (Jinan, China); Polyurethane (PU, TPUPC-3575A, Lubrizol USA); Hexafluoroisopropanol (HFIP), Shanghai Aladdin Biochemical Technology Co., Ltd (Shanghai, China); 75% ethanol disinfectant, Shandong Anjie High-Tech Disinfection Technology Co., Ltd (Dezhou, China); The silicone tube, Shanghai Liangfei Trading Co., Ltd (Shanghai, China); Heparin Sodium Injection, Shanghai Shangyao First Biochemical Pharmaceutical Co., Ltd (Shanghai, China); 0.9% sodium chloride injection, Shandong Hualu Pharmaceutical Co. Ltd (Liaocheng, China).

Experimental animals: Adult male Japanese big-eared white rabbit, weight 2.5–3.0 kg, Beijing Longan Experimental Animal Breeding Center (Beijing, China).

### Preparation of vascular scaffolds by electrospinning

**Fig. 1:**
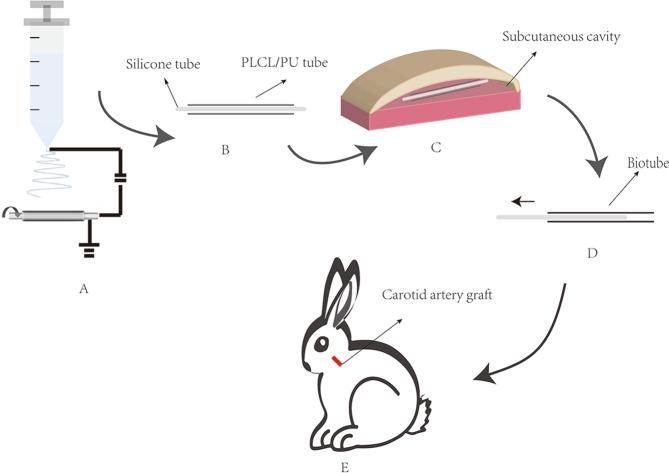
Sketch map of developing the biotube. The vascular scaffolds were prepared by electrospinning (**A**). The silicone tube was used as the core, and the vascular scaffold was used as the shell (**B**). The vascular scaffold and silicone tube were implanted subcutaneously in the abdominal area of the rabbit (**C**). The biotube was extruded from the silicone tube after 4-weeks of embedding (**D**). The biotube was implanted for the rabbit carotid artery (**E**)

**Fig. 2:**
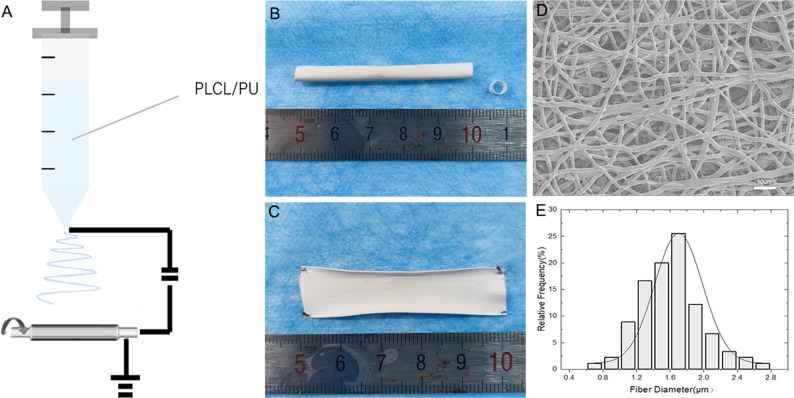
Morphology characteristics of the vascular scaffold. Schematic of the process for producing the vascular scaffold (**A**). A photograph of the vascular scaffold (**B**) and the inner surface of the scaffold (**C**). The SEM image of the electrospinning fibers (**D**). The fiber diameter distribution of the vascular scaffold (**E**)

**Fig. 3:**
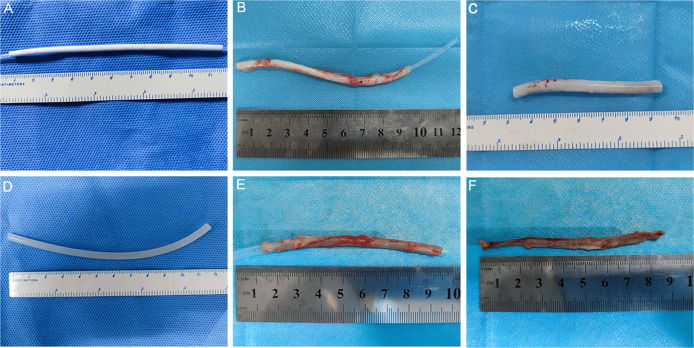
The vascular scaffold(PLCL/PU)/silicone tube(shell/core) before the embedding (**A**). After 4 weeks of embedding in the abdominal area of the rabbit (**B**). The biotube after removing the silicone tube and excess connective tissue (**C**). The silicone tube before the embedding (**D**). After 4 weeks of embedding in the abdominal area of the rabbit (**E**). The tissue capsule after removing the silicone tube (**F**)

The hybrid fibres of PLCL and PU were obtained by electrospinning of a 16% (w/v%) hybrid solution (PLCL: PU = 4: 6) in HFIP. The electrospun scaffolds were produced at a 0.3-mm/min flow rate, 8.45-kV high voltage, −0.20-kV low voltage, with19 cm between the collection point and capillary tip. The collection mandrel was 304 stainless steel bar (Teng Yiu Stainless Steel Co., China), and the rotation speed was 20 rpm (Electrospinning machine, SS-2546,Beijing Yongkang Leye Technology Development Co., Ltd). The 2.5-mm-diameter vascular scaffold (carotid artery graft) had been electrospun for 30–40 min, and the 4-mm-diameter vascular scaffold(measurement of mechanical properties) had been electrospun for 40–50 min (Figs. 1A, 2A). The thickness of the electrospun scaffold was approximately 0.35–0.40 mm. The inner surface of the fibre scaffold was observed by scanning electron microscopy (SEM) (Scanning electron microscope, Hitachi Corporation) (Fig. 2D), and the fibre diameter and surface porosity were measured and quantified by ImageJ software [10] (National Institutes of Health, USA). Then, a silicone tube was used as the core, and the vascular scaffold was used as the shell (2.5-mm-diameter vascular scaffold fitted with a 2-mm-diameter silicone tube, 4-mm-diameter vascular scaffold fitted with a 3.5-mm-diameter silicone tube). One end was secured with wire to prevent slippage (Figs. 1B, 3A).

### Subcutaneous embedding surgery

Sterilized with gamma rays (Cobalt 60 gamma ray source, 25 kGy), the vascular scaffold and silicone tube were implanted subcutaneously in the abdominal area of the rabbit (Fig. 1C). Two rods were embedded subcutaneously in each rabbit: one was a vascular scaffold/silicone tube (shell/core) (Fig. 3A), and the other was a silicone tube only (Fig. 3D). Three rabbits underwent embedding surgery. The 4-mm-diameter rods were used to measure the mechanical properties, the 2.5-mm-diameter rods were used for carotid artery grafts. A small incision was made, and two pockets were prepared. Rods were inserted into the pockets, and 5–0 sutures were used to attach to the abdominal wall. Then, the skin was sutured intermittently. The tissue capsules with the rods were harvested after four weeks. After harvesting, the biotubes and tissue capsules could easily be extruded from the silicone tube Figs. 1D, 3B, E Then, the biotubes were stored in 75% ethanol solution. Before the measurement of mechanical properties or implantation, the biotubes were washed by 0.9% sodium chloride injection for at least 10 min [9].

### Measurement of mechanical properties

The tubes were cut every 4 mm of their longitudinal length to obtain the vascular rings (*n* = 8). The thickness was measured by a spiral micrometre. Mechanical properties (circumferential) were measured by a ring tensile test using a tensile tester (DXLL5000, Shanghai Denjie Machine Equipment Co., LTD). Two triangular metal bars (external diameter: 0.9 mm) were inserted into the lumen of the ring. The bottom side was parallel and fixed on the upper and lower clamps of the tensile tester. The machine was moved at a constant speed of 2 mm/min until the sample was broken. Importing the data into Origin software (2018 version) (OriginLab Inc., USA) to generate the stress‒strain curve, we could also obtain the ultimate tensile strength (MPa), Young’s modulus (MPa), and elongation at break. Burst strength was calculated by the following Formula(1) and Formula(2) [11]. In these equations, P is the estimated internal burst pressure, F is the breaking force, L_0_ is the initial longitudinal length of the ring, Di is the effective internal diameter, d_pin_ is the diameter of the metal bar and Δs is the distance between the metal bars. The mechanical properties were displayed in GraphPad Prism 9 (GraphPad Software, Inc. USA) and analysed by one-way ANOVA (nonparametric or mixed). (Note: Biotube was formed after the embedding the PLCL/PU vascular scaffold, the tissue capsule was formed by embedding the silicone tube, the PLCL/PU vasculature was developed by electrospinning, and the native rabbit aorta was taken from the abdominal aorta of the rabbit)

1 $${{{\mathrm{P}}}} = \frac{F}{{L_0D_i}}$$

2 $$D_i = \frac{{d_{{{{\mathrm{pin}}}}}\left( {\pi + 2} \right) + 2{{\Delta }}s}}{\pi }$$

### Implantation of the rabbit carotid artery (allograft)

Adult male Japanese big-eared white rabbits (2.5–3.0 kg) were used for the experiments. All procedures were approved by the Animal Experiment Ethics Committee, Capital Medical University, China (AEEI-2017-141). Biotubes were formed by the subcutaneous embedding of the electrospun PLCL/PU vascular scaffold, and biotubes (2.5-mm diameter and 1.5-cm length) were implanted as the rabbit carotid artery (*n* = 6). As a control group, the electrospun PLCL/PU vascular scaffold was also used for rabbit carotid artery grafts (*n* = 6). Inhalation anaesthesia was administered with a 5% isoflurane mask, and the anaesthesia was maintained with 2% isoflurane. A 4-cm incision was created on the neck. After 3 min of intravenous injection of heparin sodium (100 IU/kg), both ends of the carotid artery were cross-clamped. Approximately 1.0 cm length of the artery was removed, and the vascular graft was sutured by end-to-end anastomosis with 7–0 prolene sutures. Animals received low-molecular-weight heparin (SC, 100 IU/kg, q12 h) for three days and aspirin (25 mg/QD, PO) long-term.

### Vascular ultrasound inspection

The Doppler ultrasound was performed at 1, 4, 8, and 12 weeks after carotid artery implantation (Color Doppler ultrasound machine, CX50, Philips, Netherlands). Under the anaesthetic management mentioned above, the grafts were examined by the CX50 ultrasound system, including patency, blood flow velocity, aneurysms, and thrombosis.

### Histological analysis

After four weeks of subcutaneous embedding, the biotube from the vascular scaffold/silicone tube (shell/core) and the tissue capsule from the silicone tube underwent haematoxylin and eosin (H&E) and Masson’s trichrome staining. After 4 and 12 weeks of carotid grafting, the grafts were harvested for H&E, Masson’s trichrome, and Elastica van Giessen staining. The immunofluorescence images of biotubes after 12 weeks of implantation and native rabbit aorta were stained with anti-von Willebrand Factor (vWF) (endothelial cell marker) (Servicebio; GB11020) and anti-alpha-smooth muscle actin (α-SMA) antibody (smooth muscle cell marker) (Servicebio; GB11364). The endothelial cells (ECs) and smooth muscle cells (SMC) were measured and quantified by ImageJ software (National Institutes of Health, USA).

## Results

### Electrospinning scaffold morphology characteristics

Figure 2A displays the flow diagram of the electrospinning process. The vascular scaffolds demonstrated a good shape and smooth cavity surface (Fig. 2B, C).The SEM images and fibre diameter distributions are shown in Fig. 2D, E. The fibres of the scaffolds were evenly distributed. The average diameter of the fibres was 1.7 ± 0.05 µm, and the surface porosity of the scaffolds was 40.8 ± 3.4%.

### Subcutaneous embedding of the vascular scaffold/silicone tube (shell/core) and silicone tube

The vascular scaffold/silicone tube (shell/core) was covered with connective tissue.

After four weeks of embedding, the connective tissue was tightly connected to the electrospun vascular scaffold (PLCL/PU), and the biotube could be easily removed from the silicone tube (Fig. 3B). After removing the excess connective tissue, the biotube exhibited a good shape (Fig. 3C). For the silicone tube, the tissue capsule could be easily removed (Fig. 3E), while the tissue capsule was less supportive (Fig. 3F), which was not conducive to blood vessel grafts.

**Fig. 4:**
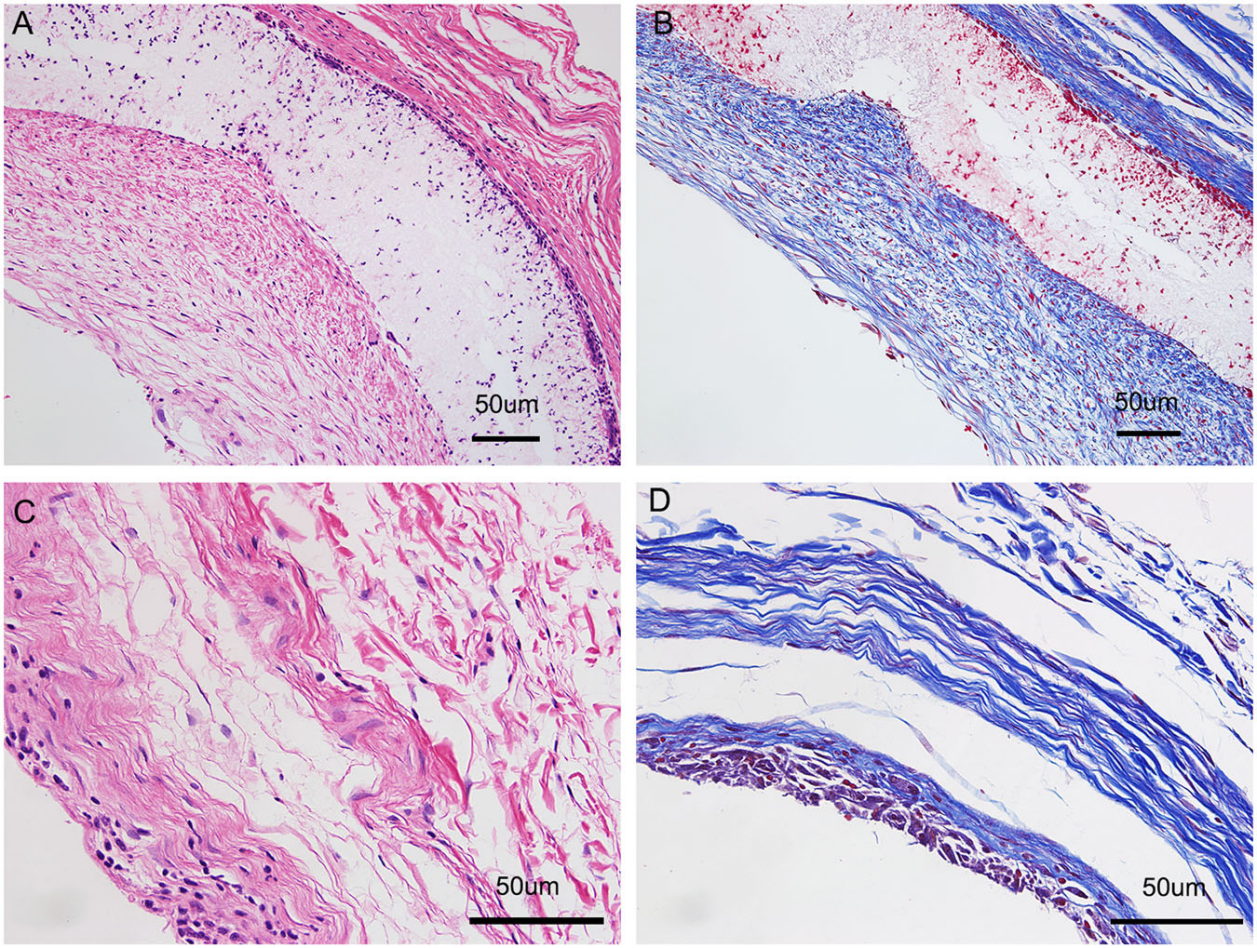
Histological cross-sectional photos of the biotube and tissue capsule. Haematoxylin and eosin (**A**) and Masson,s trichrome stain (**B**) for the biotube. Haematoxylin and eosin (**C**) and Masson,s trichrome stain (**D**) for the tissue capsule

Four weeks after the subcutaneous implantation, collagen was mainly concentrated on the inner and outer surfaces of the biotubes (Fig. 4B), and scattered cells were distributed throughout the walls of the biotubes (Fig. 4A). The tissue capsule removed from the silicone tube was mainly composed of collagen fibres (Fig. 4D) and several cellular components (Fig. 4C).

### Measurement of mechanical properties

**Fig. 5:**
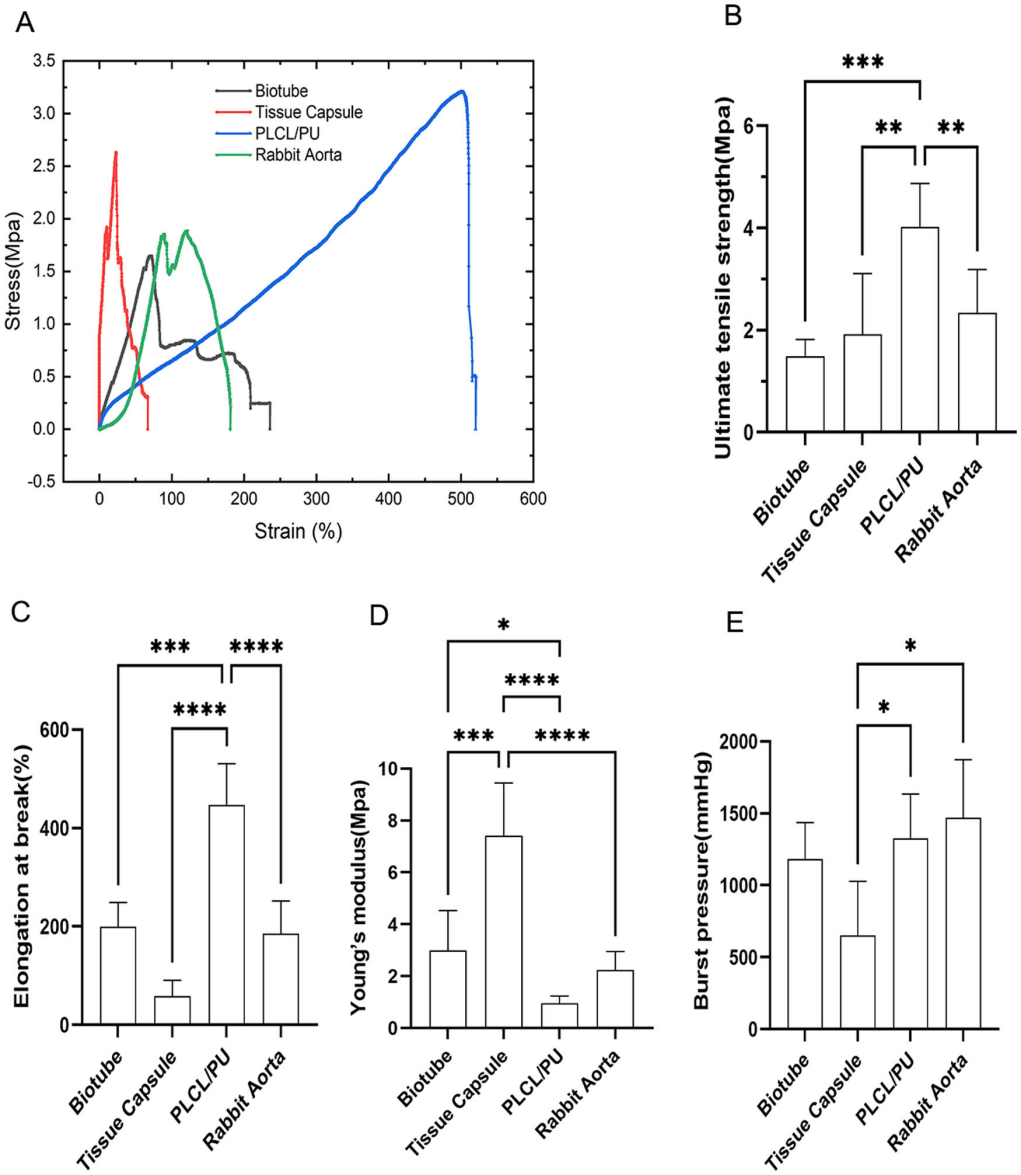
The mechanical properties (circumferential) of the biotube, tissue capsule, PLCL/PU(no embedding), and rabbit aorta. Stress-strain curve (**A**), Ultimate tensile strength (**B**), Elongation at break (**C**), Young’s modulus (**D**), Burst pressure (**E**)

The mechanical properties of the biotube (by vascular scaffold embedding), tissue capsule (by silicone tube embedding), PLCL/PU tubes (by electrospinning), and native rabbit aorta were measured. The stress‒strain curve showed a significant difference in the mechanical properties among the groups (Fig. 5A). The ultimate tensile strength of PLCL/PU (4.02 ± 0.85 MPa) was larger than that of the biotube group (1.49 ± 0.32 MPa, *P* < 0.001), tissue capsule (1.91 ± 1.19 MPa, *P* < 0.01) and rabbit aorta(2.43 ± 0.85 MPa, *P* < 0.01), although the differences were not significant between the three groups (biotube, tissue capsule and rabbit aorta groups) (Fig. 5B). For the elongation at break (Fig. 5C), the PLCL/PU group (447.3 ± 83.65%) showed superiority over the biotube (199 ± 49.57%, *P* < 0.001), tissue capsule (58.19 ± 32.46%, *P* < 0.0001), and rabbit aorta groups (185.1 ± 66.58%,*P* < 0.0001), and the three groups (biotube group, tissue capsule group and rabbit aorta group) showed no significant difference from each other (Fig. 5C). The Young’s modulus of the tissue capsule group (7.42 ± 2.02 MPa) was larger than that of the biotube (2.98 ± 1.54 MPa, *P* < 0.001), PLCL/PU (0.96 ± 0.28 MPa, *P* < 0.0001) and the rabbit aorta groups (2.25 ± 0.70Mpa, *P* < 0.0001), and the biotube group also showed a significant difference from the PLCL/PU group (*P* < 0.5) (Fig. 5D). The burst pressure of the PLCL/PU group (1327.01 ± 307.2 mmHg) was larger than that of the tissue capsule group (650.04 ± 377.5 mmHg, *P* < 0.5), the rabbit aorta group (1470.30 ± 402.1 mmHg) was also larger than that of the tissue capsule group (*P* < 0.5), and the biotube group (1183 ± 252.8 mmHg) showed no significant difference from the other groups (Fig. 5E).

### Vascular ultrasound

**Fig. 6:**
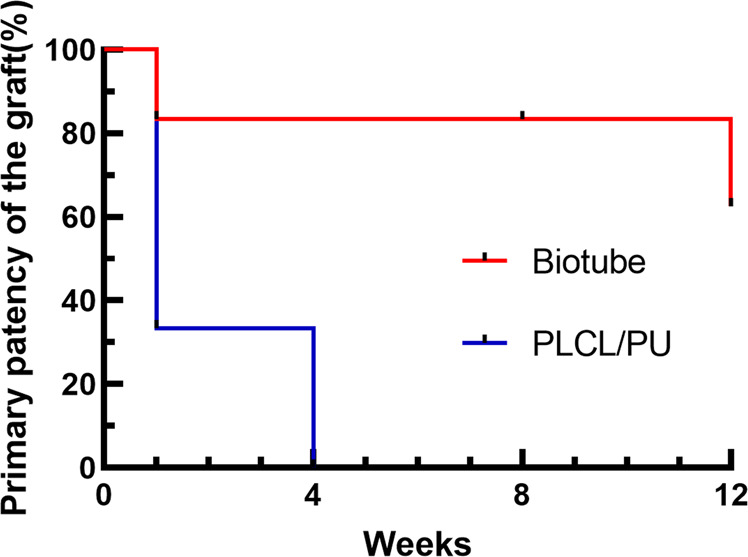
Kaplan–Meier curves showing primary patency of the grafts in the biotube group(Red line) and PLCL/PU group (Blue line)

**Fig. 7:**
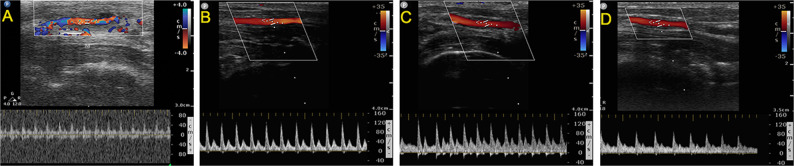
Vascular ultrasound inspection for a rabbit kept patent after implantation at 1 (**A**), 4 (**B**), 8 (**C**), and 12 weeks (**D**) in the biotube group

**Fig. 8:**
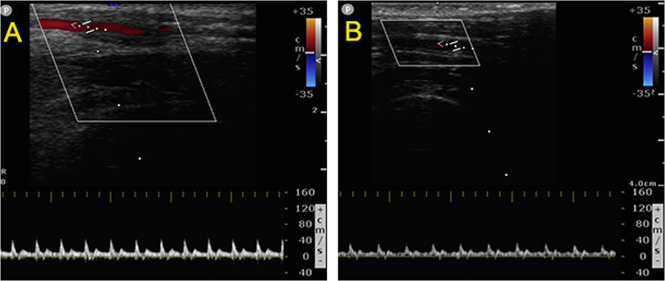
Vascular ultrasound inspection for a rabbit kept patent at 1 week (**A**) and had vascular occlusive at 4 weeks (**B**) in the PLCL/PU group

The primary patency of the biotube group was 83.33, 83.33, 83.33, and 62.50% at 1, 4, 8, and 12 weeks, respectively. In the PLCL/PU group, the primary patency was 33.33 and 0% at 1 and 4 weeks, respectively. The patency of the biotube group showed great superiority over that of the PLCL/PU group (*P* = 0.006, Table 1, Fig. 6). In the biotube group, two rabbits had the graft occlusion at 1 and 12 weeks. One rabbit died at 8 weeks (the graft remained patent after dissection), and the grafts in three rabbits remained patent at the 12-week follow-up (Fig. 7). In the PLCL/PU group, none of the grafts in the rabbits remained patent after 4 weeks of implantation (Fig. 8). During the follow-up, there was no incidence of aneurysm. Only one rabbit died in the biotube group (due to severe diarrhoea) (1/6,16.6%). In the PLCL/PU group, no rabbits died accidentally (0/6).

**Table 1:** Kaplan-Meier curves showing primary patency of the grafts in biotube group and PLCL/PU group

	1 week	4 weeks	8 weeks	12 weeks	*P* value(Log-rank)
Biotube group (6 rabbits)	83.33%	83.33%	83.33%	62.50%	0.006
PLCL/PU group (6 rabbits)	33.33%	0	–	–

### Histological observation

**Fig. 9:**
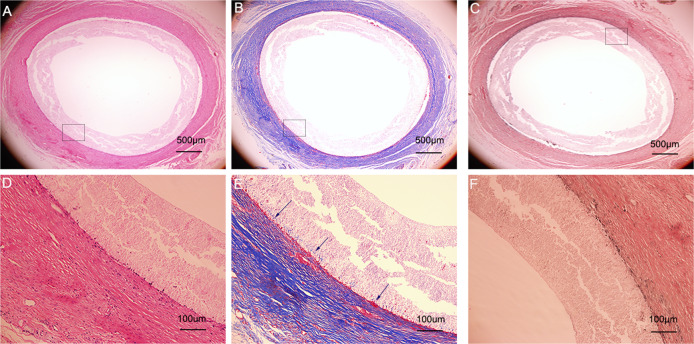
Circumferential histological images of biotube group stained with haematoxylin and eosin (**A**), Masson's trichrome (**B**), Elastica van Giessen (**C**) after 4 weeks of implantation (Partial enlarged area was labeled by the rectangle). Partial enlarged image of haematoxylin and eosin (**D**), Masson’s trichrome (**E**) (The blue part indicated by the arrow was the collagen fiber), Elastica van Giessen (**F**)

**Fig. 10:**
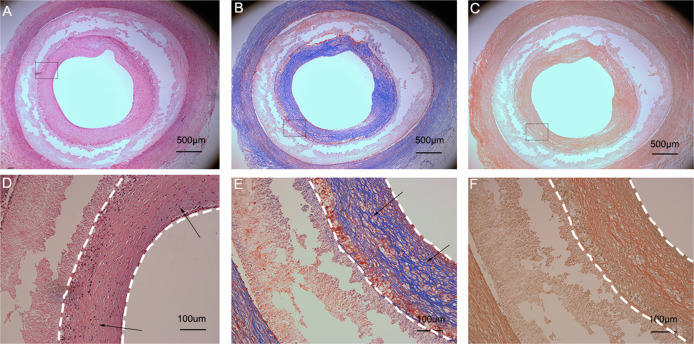
Circumferential histological images of the biotube group stained with haematoxylin and eosin (**A**), Masson's trichrome (**B**), Elastica van Giessen (**C**) after 12 weeks of implantation (Partial enlarged area was labeled by the rectangle). Partial enlarged image of haematoxylin and eosin (**D**) (The scattered nucleus were shown by the arrow), Masson's trichrome (**E**) (The blue part indicated by the arrow was the collagen fiber), Elastica van Giessen (**F**) (The white dotted areas indicated intimal hyperplasia)

**Fig. 11:**
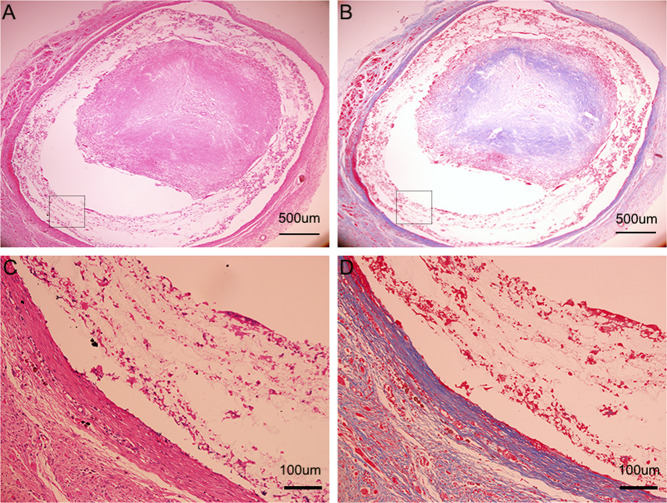
Circumferential histological images of PLCL/PU group stained with haematoxylin and eosin (**A**), Masson's trichrome (**B**) after 4 weeks implantation (Partial enlarged area was labeled by the rectangle). Partial enlarged image of haematoxylin and eosin (**C**), Masson's trichrome (**D**)

**Fig. 12:**
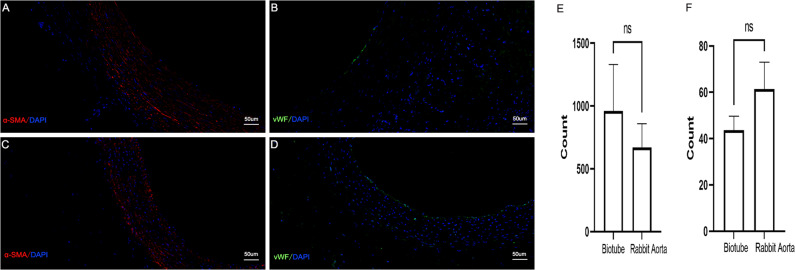
The Immunofluorescence images of biotubes after 12 weeks of implantation (**A**, **B**) and native rabbit aorta (**C**, **D**) stained withα-SMA (**A**, **C** positive-Red, DAPI-Blue), vWF (**B**, **D**, positive-Green, DAPI-Blue). The α-SMA positive cells (**E**) and vWF positive cells (**F**) were quantified

Circumferential histological images of the biotube group stained with haematoxylin and eosin, Masson’s trichrome, and Elastica van Giessen after 4 (Fig. 9) and 12 weeks (Fig. 10) following implantation. As the implantation time increased, intimal hyperplasia occurred (Fig. 10, white dotted area), and there were no obvious elastic fibres (Figs. 9C, F, 10C, F). In the PLCL/PU group, the haematoxylin and eosin and Masson’s trichrome histological images showed apparent occlusion. The graft’s luminal surface was rough, and collagen fibres were not present in the graft (Fig. 11). The immunofluorescence images of α-SMA and vWF for the biotube graft (after 12 weeks of implantation) and the native rabbit aorta were confirmed and quantified, as shown in Fig. 12. The regular and uniform SMC structures of the biotube graft (Fig. 12A) were similar to those of the native rabbit aorta (Fig. 12B), while the cell count of the SMC cells in the biotube graft was larger than that in the native rabbit aorta (960 ± 370.3 vs 671.7 ± 187.4, *P* = 0.29) (Fig. 12E). The endothelial cell lining of the biotube graft (Fig. 12C) was similar to that of the native rabbit aorta (Fig. 12D), while the count of the ECs in the biotube graft was lower than that of the native rabbit aorta (43.67 ± 6.0 vs. 61.33 ± 11.7, *P* = 0.08) (Fig. 12F). These differences in the cell count in SMCs and ECs were not statistically significant.

## Discussion

This study developed a new method combining electrospinning and in-body tissue architecture (iBTA) to develop small-calibre TEBVs. The fibres produced by electrospinning are similar to the physical structure of collagen fibres in the ECM, which are beneficial for cell proliferation and development [12]. The technology can produce vascular scaffolds with different properties according to the use of various polymers. We chose PLCL and PU to develop vascular scaffolds in this study. PLCL is a copolymer of lactic acid and caprolactone. In the copolymer, the brittle behaviour of polylactic acid (PLA) and the low level of stiffness of polycaprolactone (PCL) are adjusted [13]. PLCL shows good elasticity and biocompatibility and slow biodegradation [14]. In a rabbit animal model, the degradation rate of PLCL was approximately six months in vivo [15]. PU is well known for its mechanical properties and appropriate biocompatibility, especially for its high tensile strength and toughness, making it one of the most important synthetic polymers for the fabrication of vascular grafts [16]. We obtained hybrid fibres (PLCL/PU) by electrospinning the hybrid solution. To further enhance the graft’s mechanical strength, we appropriately increased the proportion of PU (PLCL: PU = 4:6). PLCL degradation gave the cells and tissues more space to grow and proliferate. The PU ensured the mechanical properties of vascular scaffolds. The fibres of the scaffolds were evenly distributed via SEM observation. The average diameter of the fibres was 1.7 ± 0.05 µm, and the porosity of the scaffolds was 40.8 ± 3.4%. An electrospun scaffold with microscale porous structures can provide nutrients, cellular infiltration, and gas exchange, which are crucial for tissue regeneration and cell viability [17].

The lack of biological properties is a major limitation of vascular scaffolds developed by synthetic polymers, resulting in incomplete endothelization, stenosis, and thrombosis after implantation [4]. These limitations can be reduced to some extent by bioactive molecules (vascular endothelial growth factor, heparin [18], chondroitin sulfate [19]) and natural polymers (e.g., gelatine, collagen, chitosan, elastin, lecithin, silk fibroin [18]). The majority of these methods involve complex in vitro preparation steps and are costly. IBTA provides a novel option for improving the biological properties of vascular scaffolds. After subcutaneous embedding, the vascular scaffolds developed by electrospinning were surrounded by collagen. At the same time, the scaffolds provided some collagen support. Therefore, the shape of the biotube could be well maintained. The iBTA was based on the foreign body response (FBR). In the early stage of the FBR, the main components of the tissue capsule were neutrophils and less collagen (with poor mechanical properties). However, an embedding time that is too long (several months) was not conducive to vascular remodelling for the largely acellular tissue [5]. Several studies on the subcutaneous embedding showed that 4 weeks was the appropriate time [9, 20, 21], and the tissue capsule contained sufficient collagen and exhibited good vascular remodelling after 4 weeks of embedding. Therefore, the embedding time was also 4 weeks in this study.

The ultimate tensile strength (circumferential) of the biotube was 1.49 ± 0.32 MPa, similar to that of the saphenous vein (1.8 MPa) [22], which was smaller than that of PLCL/PU (4.02 ± 0.85 MPa, *P* < 0.001) due to the partial degradation of PLCL. Although the ultimate tensile strength of the biotube was smaller than that of tissue capsule (1.91 ± 1.19 Mpa) and the rabbit aorta (2.43 ± 0.85 Mpa), there was no significant difference. The elongation at the break of the tissue capsule (58.19 ± 32.46%) was particularly poor. The biotube group (199 ± 49.57%) showed similar elongation to the rabbit aorta (185.1 ± 66.58%), and the PLCL/PU group (447.3 ± 83.65%) showed superiority over the other three groups. The Young’s modulus of the tissue capsule (7.42 ± 2.02 MPa) was the largest among the four groups, which suggested that the tissue capsule was not prone to deformation, while that of the PLCL/PU group (0.96 ± 0.28 MPa) was too low. The Young’s modulus of the biotube group (2.98 ± 1.54 MPa) was similar to that of the rabbit aorta (2.25 ± 0.70 MPa), which was closest to that of the coronary artery (1.41 ± 0.72 MPa) [23]. For the burst pressure, the PLCL/PU (1327.01 ± 307.2 mmHg), the rabbit aorta (1470.30 ± 402.1 mmHg), and the biotube groups (1183 ± 252.8 mmHg) showed no significant difference from each other. The results were similar to that of the saphenous vein (1599 ± 877 mmHg) [24], while the tissue capsule (650.04 ± 377.5 mmHg) showed a worse result. During the follow-up, none of the rabbits had aneurysms. In summary, the mechanical strength of the PLCL vasculature is relatively strong, but the elasticity deformation (Young’s modulus) differed significantly from that of the native rabbit carotid artery. The overall mechanical strength of the tissue capsule was relatively low. The mechanical properties of the biotube were most similar to those of the native rabbit aorta, mainly because the biotube integrates the mechanical properties of the PLCL/PU vascular scaffold and tissue capsule through iBTA technology and partial degradation of PLCL.

During implantation, the biotube was easy to suture onto native vessels. The tube wall would not ooze blood after releasing the haemostatic clamp. While the PLCL/PU tube wall would ooze after releasing the clamp, the bleeding would stop after the pressure with gauze for approximately three minutes. This may be related to the existence of collagen in the biotube wall. During the follow-up, the patency of the biotube group showed great superiority over that of the PLCL/PU group. The grafts in the PLCL/PU group were all occlusive within 4 weeks, while the primary patency in the biotube group was 62.5% after 12 weeks of implantation. In other words, the iBTA resulted in a higher patency for the PLCL/PU tube, which was related to the existence of ECM components. The native vascular ECM comprises collagens, laminins, elastins, filamentous proteins, glycosaminoglycans (GAGs), and proteoglycans, which play important roles in cellular function and tissue architecture [25]. Collagens provide tensile strength for the vascular walls [26]. GAGs include cytokines and growth factors, and they are also related to the viscoelasticity [27]. Vitronectin and fibronectin are related to interactions between the ECM and cells through integrins, which are essential to vascular remodelling [28]. Thus, many studies of small-calibre TEBVs are related to the vascular ECM. Decellularization is the primary method to obtain ECM vascular scaffolds by using native vessels, such as the carotid artery, aortic artery, and internal mammary artery. The main methods for vessel decellularization include enzymes, surfactants, solvents, and physical methods [4]. Arginine-glycine-aspartic acid (RGD), a tripeptide derived from the cell adhesion sequence of fibronectin (the main component of the ECM), has been proven to promote the endothelialization and patency of vascular grafts [29]. Collagen type IV (COL IV) is the main component of the vascular basement membrane (VBM), a thin layer of fibrous ECM linking the endothelium, which also improves the endothelialization of the vascular grafts [30]. The ECM is similar to a “black box”, and we cannot fully understand the components and biological functions of vascular grafts; however, through the “black box”, vascular grafts “output” better results. Compared with other methods, iBTA is more convenient and economical for delivering ECM to the vascular grafts.

In the biotube group, compared with the histological examination after 4 weeks of implantation, intimal hyperplasia appeared after 12 weeks of implantation. Although the grafts remained patent, intimal hyperplasia was a risk factor for decreased long-term patency. The main reasons included the excessive proliferation of smooth muscle cells, undistributed graft wall stress and compliance mismatch [31], especially at the anastomosis site. In this study, intimal hyperplasia was also mainly present at the proximal and distal anastomoses of the biotube. Therefore, rabbits require long-term observation to determine whether intimal hyperplasia has progressed. Several studies have suggested that heparin-bonded grafts have an anticoagulant effect and reduce anastomotic intimal hyperplasia [32, 33]. Heparin plays a vital role in modulating neointimal proliferation and smooth muscle cell migration via a complex biochemical and cellular cascade, which can inhibit the deoxyribonucleic acid (DNA) synthesis and downregulate the transcription of genes in smooth muscle cells [34]. During the signal transduction of smooth muscle cells, heparin can downregulate transcription activator proteins [35] and inhibit intracellular protein kinase activity [36]. Therefore, in future studies, we plan to combine heparin and biotubes to inhibit neointimal proliferation and increase long-term patency.

After 12 weeks of implantation, α-SMA-positive cells were observed in the biotube and arranged in layers. vWF-positive cells were observed in the lining in of the luminal surface. The SMC structures and the endothelial cell lining of the biotube graft were similar to those of the native rabbit aorta. The cell count of the SMCs cells in the biotube graft was larger than that in the native rabbit aorta (no statistical significance), which may be related to the intimal hyperplasia of the biotube graft, while the cell count of the ECs in the biotube graft was less than that in the native rabbit aorta (no statistical significance), which was related to the incomplete endothelialization of the biotube graft. The results suggested that the biotube was capable of remodelling, and long-term observation was still needed. Blood flow and cyclic stretch are potent stimuli for ECM synthesis and remodelling, and they also promote (myo)fibroblast differentiation into vascular smooth muscle cells [37, 38]. Endothelial cells (ECs) are vital in antithrombogenicity by suppressing platelet activation and subsequent coagulation cascades [39]. However, the origin of the ECs is controversial, and the endothelialization of the grafts may arise from circulating endothelial progenitor cells [40] or result from the migration of ECs from neighbouring vessels [5]. The necessity of an endothelial lining in TEBVs before vascular implantation is controversial. The collagen was distributed throughout part of the lumen surface of the biotube,with no ECs lining the tube, thus resulting in intrinsic thrombogenicity [41]. However, no acute thrombosis was observed except for anastomotic stenosis in this study. Despite the fact that most researchers aim to make their vascular scaffolds endothelial, some studies revealed that TEVGs with no or little initial endothelial coverage had excellent long-term patency in large [42] and small [43] animal models. These studies further illustrate the feasibility of using biotubes with collagen and no endothelial coverage as TEVGs.

Before the allograft, the biotubes were stored in 75% ethanol to kill the cells and reduce the immune response. This method has been proven to be effective in other research [9]. While the cellular components that remain in the biotube may still induce an immune response, decellularization provides us new options to minimize the cell-derived adverse effects. This method can remove cellular and antigen material through surfactants, enzymes, solvents, and physical methods, reducing the risk of immune and inflammatory responses [27]. Decellularization will be included in future studies.

## Conclusion

In this study, we created a new method that combines electrospinning and iBTA to develop small-calibre TEBVs. Electrospinning imparted mechanical properties to the TEBVs, and iBTA imparted biological properties to the the TEBVs. The mechanical properties of the biotubes were most similar to those of the native rabbit aorta. The biotube exhibited a better patency rate and certain remodelling ability in the rabbit model, which indicated the potential use of this hybridization method to develop small-calibre TEBVs.
